# Supplementary figures and images for: Bleaching-driven reef community shifts drive pulses of increased reef sediment generation
Source: R Soc Open Sci. 2020 Apr 22;7(4):192153. doi: 10.1098/rsos.192153 (PMC7211869; doi:10.1098/rsos.192153)

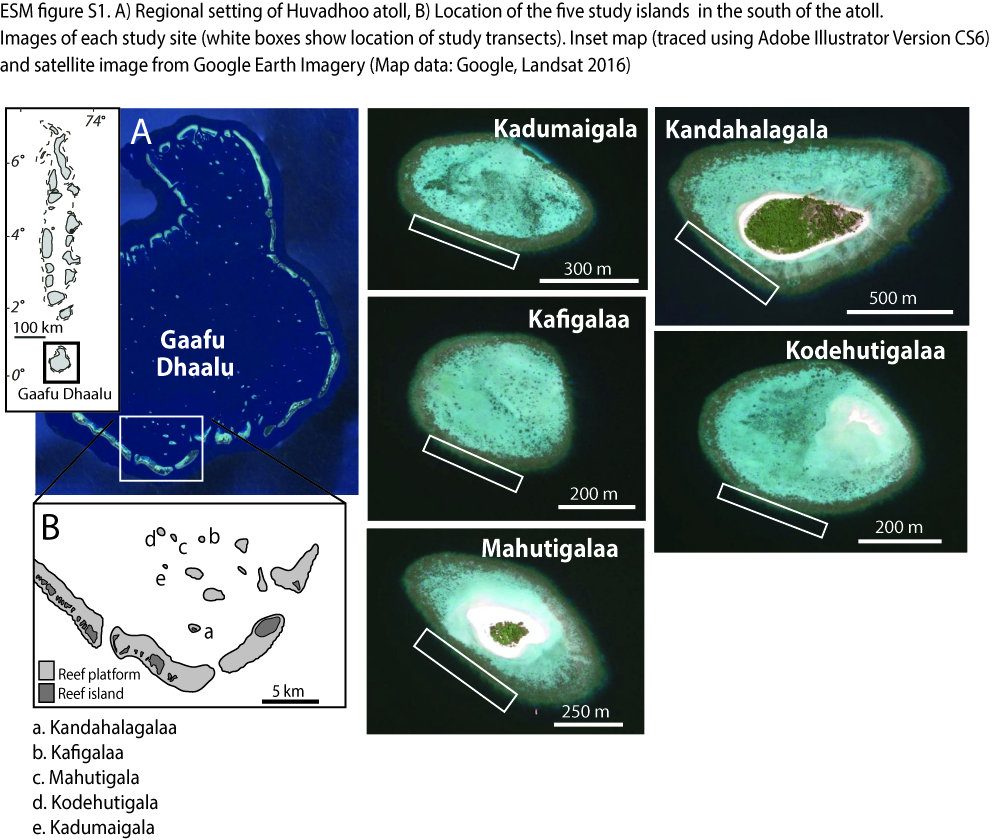

Supplement: Fig S1 [file rsos192153supp1.tif]

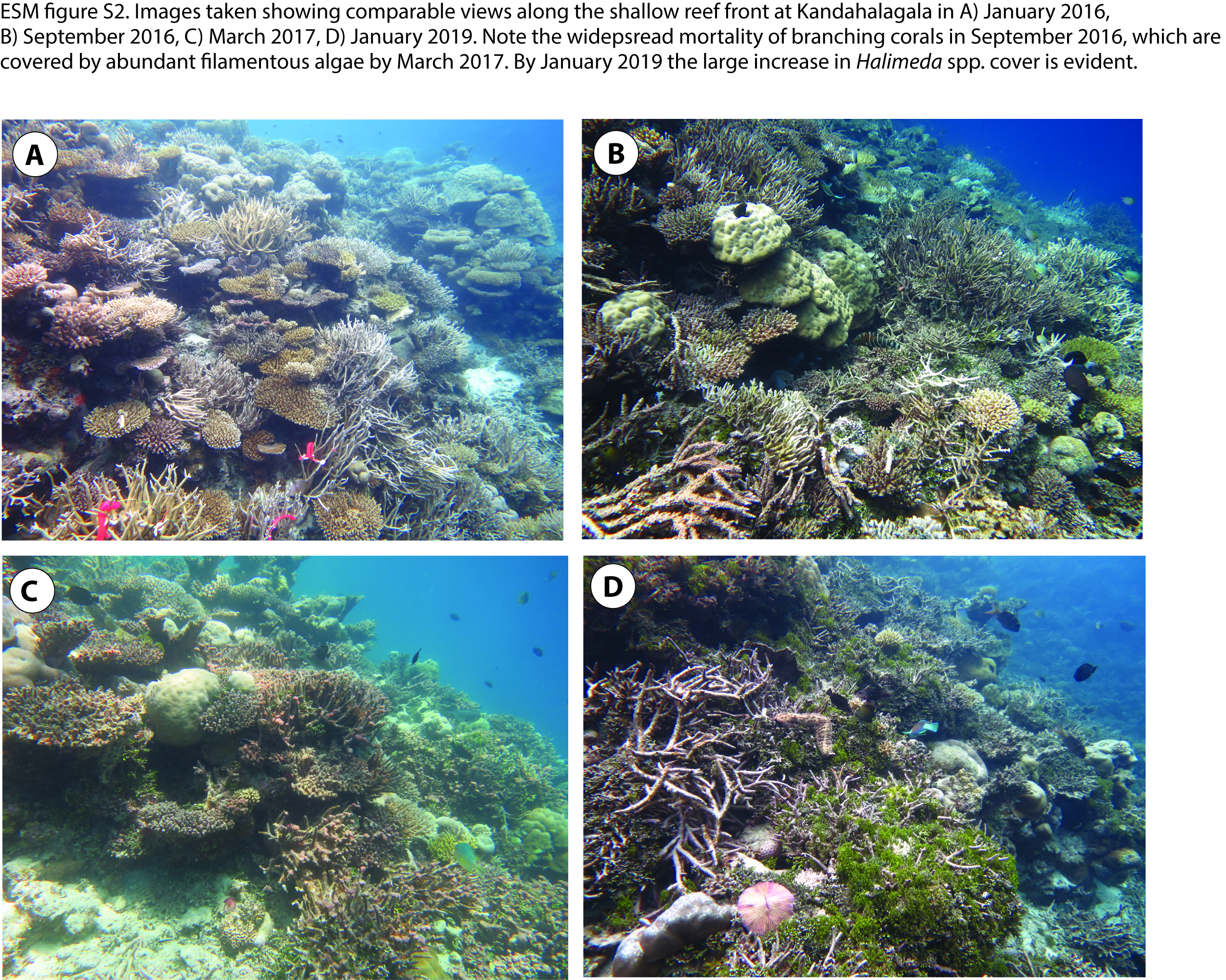

Supplement: Fig S2 [file rsos192153supp2.tif]

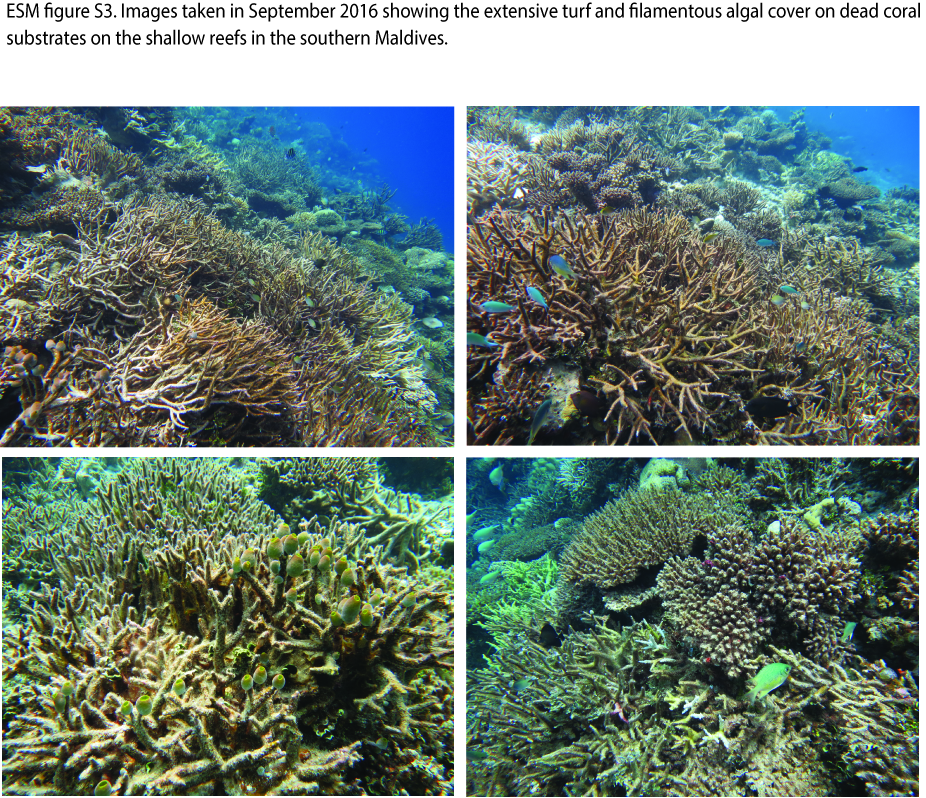

Supplement: Fig S3 [file rsos192153supp3.tif]

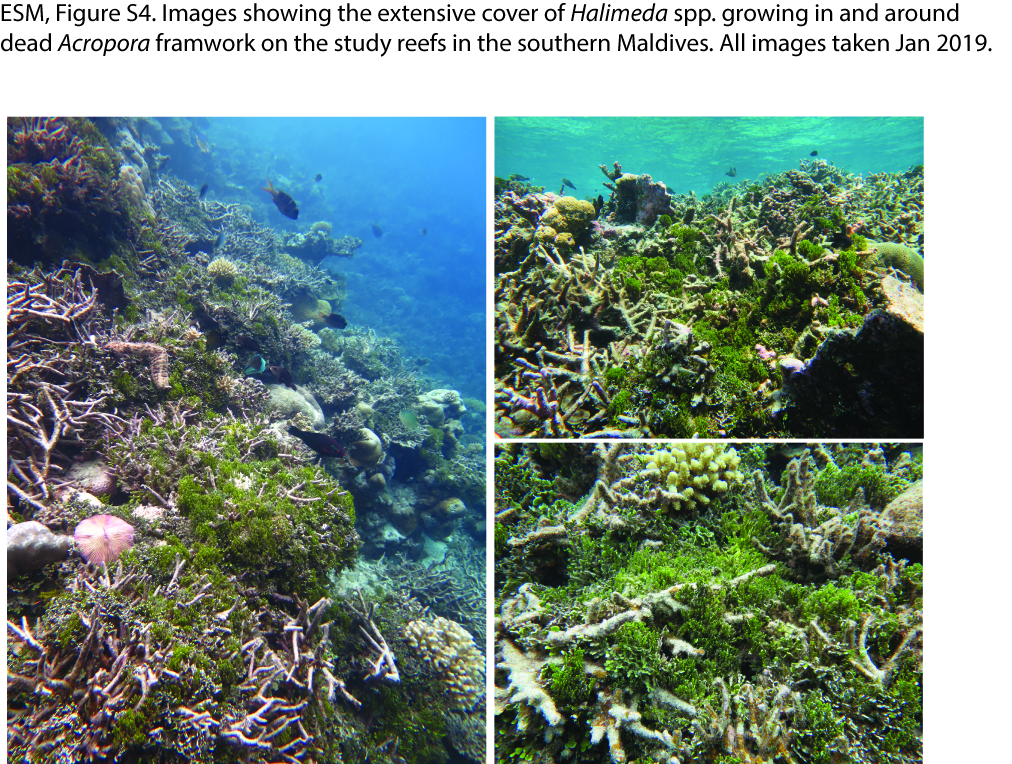

Supplement: Fig S4 [file rsos192153supp4.tif]
