# Supplementary material for: Bleaching-driven reef community shifts drive pulses of increased reef sediment generation
Source: R Soc Open Sci. 2020 Apr 22;7(4):192153. doi: 10.1098/rsos.192153 (PMC7211869; doi:10.1098/rsos.192153)
Supplement: Table S1 [file rsos192153supp5.doc]

**ESM Table S3. Results of paired t-tests comparing parrotfish biomass, benthic ecology and parrotfish and *Halimeda* spp. sediment production across the January 2016 to March 2017 sampling periods**

Results comparing mean parrotfish biomass (kg ha^-1^) between January 2016 and September 2016.

| **Variable** | **January 2016** | | **September 2016** | |  | | |
| --- | --- | --- | --- | --- | --- | --- | --- |
|  | **Mean** | **SD** | **Mean** | **SD** | ***t(4)*** | ***p*** | **95% Cl** |
| Total | 126.74 | 10.79 | 256.00 | 42.18 | 6.6371 | 0.0002 | -174.17, -84.35 |
| H. harid | 2.23 | 3.53 | 12.44 | 8.03 | 2.5998 | 0.0316 | -19.26, -1.15 |
| C. ocellatus | 12.08 | 10.99 | 28.96 | 6.63 | 2.9396 | 0.0187 | -30.12, -3.63 |
| C. sordidus | 53.55 | 13.21 | 79.02 | 17.74 | 2.5746 | 0.0329 | -48.28, -2.65 |
| C. strongylocephalus | 19.55 | 7.71 | 76.83 | 29.83 | 4.1575 | 0.0032 | -89.06, -25.51 |
| S. frenatus | 1.21 | 2.30 | 0.15 | 0.34 | 1.0155 | 0.3396 | -1.34, 3.46 |
| S. niger | 18.36 | 3.76 | 33.03 | 13.21 | 2.3877 | 0.0440 | -28.83, -0.50 |
| S. psittacus | 17.21 | 4.70 | 19.38 | 10.46 | 0.4237 | 0.6829 | -13.99, 9.65 |
| S. tricolor | 1.71 | 1.79 | 3.85 | 2.60 | 1.5152 | 0.1682 | -5.39, 1.11 |

Results comparing mean parrotfish biomass (kg ha^-1^) between September 2016 and March 2017.

| **Variable** | **September 2016** | | **March 2017** | |  | | |
| --- | --- | --- | --- | --- | --- | --- | --- |
|  | **Mean** | **SD** | **Mean** | **SD** | ***t(4)*** | ***p*** | **95% Cl** |
| Total | 256.00 | 42.18 | 263.96 | 23.35 | 0.3686 | 0.7220 | -57.67, 41.78 |
| H. harid | 12.44 | 8.03 | 7.29 | 10.82 | 0.8547 | 0.4176 | -8.74, 19.05 |
| C. ocellatus | 28.96 | 6.63 | 37.71 | 24.19 | 0.7794 | 0.4582 | -34.61, 17.12 |
| C. sordidus | 79.02 | 17.74 | 88.90 | 28.54 | 0.6574 | 0.5294 | -44.54, 24.78 |
| C. strongylocephalus | 76.83 | 29.83 | 71.62 | 25.89 | 0.2955 | 0.7752 | -35.52, 45.96 |
| S. frenatus | 0.15 | 0.34 | 1.64 | 1.92 | 1.7036 | 0.1269 | -3.51, 0.52 |
| S. niger | 33.03 | 13.21 | 33.28 | 9.62 | 0.0335 | 0.9741 | -17.10, 16.61 |
| S. psittacus | 19.38 | 10.46 | 14.48 | 7.36 | 0.8558 | 0.4170 | -8.29, 18.08 |
| S. tricolor | 3.85 | 2.60 | 3.23 | 3.18 | 0.3367 | 0.7450 | -3.62, 4.85 |

Results comparing mean parrotfish biomass (kg ha^-1^) between March 2017 and Jan 2019.

| **Variable** | **March 2017** | | **January 2019** | |  | | |
| --- | --- | --- | --- | --- | --- | --- | --- |
|  | **Mean** | **SD** | **Mean** | **SD** | ***t(4)*** | ***p*** | **95% Cl** |
| Total | 263.96 | 23.35 | 354.81 | 124.47 | 1.6043 | 0.1473 | -221.46, 39.74 |
| H. harid | 7.29 | 10.82 | 17.02 | 21.44 | 0.9062 | 0.3913 | -34.50, 15.03 |
| C. ocellatus | 37.71 | 24.19 | 68.95 | 47.18 | 1.3177 | 0.2241 | -85.92, 23.43 |
| C. sordidus | 88.90 | 28.54 | 86.67 | 17.77 | 0.1483 | 0.8858 | -32.44, 36.90 |
| C. strongylocephalus | 71.62 | 25.89 | 93.72 | 78.03 | 0.6011 | 0.5644 | -106.88, 62.68 |
| S. frenatus | 1.64 | 1.92 | 1.91 | 2.91 | 0.1709 | 0.8686 | -3.87, 3.33 |
| S. niger | 33.28 | 9.62 | 28.69 | 7.35 | 0.8464 | 0.4219 | -7.90, 17.08 |
| S. psittacus | 14.48 | 7.36 | 10.87 | 9.35 | 0.6782 | 0.5168 | -8.66, 15.88 |
| S. tricolor | 3.23 | 3.18 | 7.67 | 5.88 | 1.4848 | 0.1759 | -11.33, 2.45 |

Results comparing mean coral cover, turf algal cover and *Halimeda* cover between Jan 2016 and Sept 2016

| **Variable** | **January 2016** | | **Sept 2016** | |  | | |
| --- | --- | --- | --- | --- | --- | --- | --- |
|  | **Mean** | **SD** | **Mean** | **SD** | ***t(48)*** | ***p*** | **95% Cl** |
| Coral | 25.56 | 8.04 | 4.19 | 2.66 | 12.6013 | 0.0001 | 17.96, 24.78 |
| Turf | 14.72 | 5.67 | 25.75 | 9.17 | 5.1088 | 0.0001 | -15.36, -6.68 |
| *Halimeda* | 6.43 | 3.45 | 5.33 | 1.95 | 1.3833 | 0.1730 | -0.49, 2.69 |

Results comparing mean coral cover, turf algal cover and *Halimeda* cover between Sept 2016 and March 2017

| **Variable** | **Sept 2016** | | **March 2017** | |  | | |
| --- | --- | --- | --- | --- | --- | --- | --- |
|  | **Mean** | **SD** | **Mean** | **SD** | ***t(48)*** | ***p*** | **95% Cl** |
| Coral | 4.19 | 2.66 | 4.72 | 3.22 | 0.631 | 0.5271 | -2.21, 1.15 |
| Turf | 25.75 | 9.17 | 27.46 | 10.34 | 0.6166 | 0.5404 | -7.26, 3.85 |
| *Halimeda* | 5.33 | 1.95 | 4.87 | 4.11 | 0.5069 | 0.6145 | -1.36, 2.29 |

Results comparing mean coral cover, turf algal cover and *Halimeda* cover between March 2017 and January 2019

| **Variable** | **March 2017** | | **January 2019** | |  | | |
| --- | --- | --- | --- | --- | --- | --- | --- |
|  | **Mean** | **SD** | **Mean** | **SD** | ***t(48)*** | ***p*** | **95% Cl** |
| Coral | 4.72 | 3.22 | 8.79 | 4.72 | 3.5521 | 0.0009 | -6.36, -1.76 |
| Turf | 27.46 | 10.34 | 41.66 | 13.82 | 4.1119 | 0.0002 | -21.14, -7.25 |
| *Halimeda* | 4.87 | 4.11 | 20.46 | 8.36 | 8.3637 | 0.0001 | -19.33, -11.84 |

Results comparing mean parrotfish bioerosion/sediment production (kg CaCO_3_ m^-2^ yr^-1^) between January 2016 and September 2016.

| **Variable** | **January 2016** | | **September 2016** | |  | | |
| --- | --- | --- | --- | --- | --- | --- | --- |
|  | **Mean** | **SD** | **Mean** | **SD** | ***t(4)*** | ***p*** | **95% Cl** |
| Total | 0.47 | 0.15 | 1.97 | 0.49 | 6.4262 | 0.0002 | -2.04, -0.96 |
| H. harid | 0.00 | 0.00 | 0.00 | 0.00 | 2.5755 | 0.0328 | -0.00, 0.00 |
| C. ocellatus | 0.05 | 0.06 | 0.23 | 0.08 | 3.7765 | 0.0054 | -0.27, -0.07 |
| C. sordidus | 0.17 | 0.04 | 0.24 | 0.06 | 2.2566 | 0.0540 | -0.14, 0.00 |
| C. strongylocephalus | 0.19 | 0.13 | 1.44 | 0.46 | 5.7609 | 0.0004 | -1.73, -0.74 |
| S. frenatus | 0.00 | 0.00 | 0.00 | 0.00 | 1.0050 | 0.3443 | -0.00, 0.00 |
| S. niger | 0.01 | 0.00 | 0.02 | 0.02 | 1.6367 | 0.1403 | -0.02, 0.00 |
| S. psittacus | 0.03 | 0.01 | 0.03 | 0.02 | 0.0924 | 0.9287 | -0.03, 0.02 |
| S. tricolor | 0.00 | 0.00 | 0.00 | 0.00 | 1.4726 | 0.1791 | -0.00, 0.00 |

Results comparing mean parrotfish bioerosion/sediment production (kg CaCO_3_ m^-2^ yr^-1^) between September 2016 and March 2017.

| **Variable** | **September 2016** | | **March 2017** | |  | | |
| --- | --- | --- | --- | --- | --- | --- | --- |
|  | **Mean** | **SD** | **Mean** | **SD** | ***t(4)*** | ***p*** | **95% Cl** |
| Total | 1.97 | 0.49 | 2.11 | 0.52 | 0.4413 | 0.6707 | -0.89, 0.60 |
| H. harid | 0.00 | 0.00 | 0.00 | 0.00 | 0.8014 | 0.4461 | -0.00, 0.00 |
| C. ocellatus | 0.23 | 0.08 | 0.47 | 0.45 | 1.1883 | 0.2688 | -0.72, 0.23 |
| C. sordidus | 0.24 | 0.06 | 0.26 | 0.09 | 0.4110 | 0.6919 | -0.13, 0.09 |
| C. strongylocephalus | 1.44 | 0.46 | 1.31 | 0.38 | 0.4637 | 0.6552 | -0.49, 0.74 |
| S. frenatus | 0.00 | 0.00 | 0.00 | 0.00 | 1.6236 | 0.1431 | -0.00, 0.00 |
| S. niger | 0.02 | 0.02 | 0.02 | 0.01 | 0.4369 | 0.6737 | -0.02, 0.01 |
| S. psittacus | 0.03 | 0.02 | 0.02 | 0.01 | 1.0423 | 0.3277 | -0.01, 0.03 |
| S. tricolor | 0.00 | 0.00 | 0.00 | 0.00 | 0.4912 | 0.6365 | -0.00, 0.00 |

Results comparing mean parrotfish bioerosion/sediment production (kg CaCO_3_ m^-2^ yr^-1^) between March 2017 and January 2019.

| **Variable** | **March 2017** | | **January 2019** | |  | | |
| --- | --- | --- | --- | --- | --- | --- | --- |
|  | **Mean** | **SD** | **Mean** | **SD** | ***t(4)*** | ***p*** | **95% Cl** |
| Total | 2.11 | 0.52 | 3.47 | 1.91 | 1.5285 | 0.1649 | -3.39, 0.68 |
| H. harid | 0.00 | 0.00 | 0.01 | 0.01 | 0.9889 | 0.3517 | -0.02, 0.01 |
| C. ocellatus | 0.47 | 0.45 | 1.27 | 0.86 | 1.8269 | 0.1051 | -1.80, 0.20 |
| C. sordidus | 0.26 | 0.09 | 0.34 | 0.07 | 1.4338 | 0.1895 | -0.19, 0.04 |
| C. strongylocephalus | 1.31 | 0.38 | 1.73 | 1.51 | 0.5909 | 0.5709 | -2.02, 1.20 |
| S. frenatus | 0.00 | 0.00 | 0.00 | 0.00 | 0.4046 | 0.6964 | -0.00, 0.00 |
| S. niger | 0.02 | 0.01 | 0.02 | 0.01 | 0.3754 | 0.7172 | -0.01. 0.02 |
| S. psittacus | 0.02 | 0.01 | 0.02 | 0.02 | 0.3470 | 0.7376 | -0.03, 0.02 |
| S. tricolor | 0.00 | 0.00 | 0.01 | 0.01 | 1.2754 | 0.2380 | -0.01, 0.00 |

Results comparing mean *Halimeda* production between sampling periods

| **Variable** | **January 2016** | | **September 2016** | |  | | |
| --- | --- | --- | --- | --- | --- | --- | --- |
|  | **Mean** | **SD** | **Mean** | **SD** | ***t(48)*** | ***p*** | **95% Cl** |
| *Halimeda* | 0.07 | 0.04 | 0.06 | 0.02 | 1.790 | 0.0798 | -1.91, 32.89 |

| **Variable** | **September 2016** | | **March 2017** | |  | | |
| --- | --- | --- | --- | --- | --- | --- | --- |
|  | **Mean** | **SD** | **Mean** | **SD** | ***t(48)*** | ***p*** | **95% Cl** |
| *Halimeda* | 0.06 | 0.02 | 0.05 | 0.04 | 0.5069 | 0.6145 | -15.51, 25.98 |

| **Variable** | **March 2017** | | **January 2019** | |  | | |
| --- | --- | --- | --- | --- | --- | --- | --- |
|  | **Mean** | **SD** | **Mean** | **SD** | ***t(48)*** | ***p*** | **95% Cl** |
| *Halimeda* | 0.05 | 0.04 | 0.23 | 0.09 | 8.3637 | 0.0001 | -219.30, -134.29 |
